# The Effect of Fermentation with Kefir Grains on the Physicochemical and Antioxidant Properties of Beverages from Blue Lupin (*Lupinus angustifolius* L.) Seeds

**DOI:** 10.3390/molecules25245791

**Published:** 2020-12-08

**Authors:** Łukasz Łopusiewicz, Emilia Drozłowska, Paulina Trocer, Paweł Kwiatkowski, Artur Bartkowiak, Annett Gefrom, Monika Sienkiewicz

**Affiliations:** 1 Center of Bioimmobilisation and Innovative Packaging Materials, Faculty of Food Sciences and Fisheries, West Pomeranian University of Technology Szczecin, Janickiego 35, 71-270 Szczecin, Poland; emilia_drozlowska@zut.edu.pl (E.D.); p.trocer@gmail.com (P.T.); Artur-Bartkowiak@zut.edu.pl (A.B.); 2 Department of Diagnostic Immunology, Chair of Microbiology, Immunology and Laboratory Medicine, Pomeranian Medical University in Szczecin, Powstańców Wielkopolskich 72, 70-111 Szczecin, Poland; pawel.kwiatkowski@pum.edu.pl; 3 Mecklenburg-Vorpommern Research Centre for Agriculture and Fisheries, Dorfplatz 1/OT Gülzow, 18276 Gülzow-Prüzen, Germany; a.gefrom@lfa.mvnet.de; 4 Department of Allergology and Respiratory Rehabilitation, Medical University of Łódź, Żeligowskiego 7/9, 90-752 Łódź, Poland; monika.sienkiewicz@umed.lodz.pl

**Keywords:** lupin, vegan foods, functional foods, fermentation, kefir, lactic acid bacteria, yeast, antioxidants, free radicals scavenging

## Abstract

Plant derived fermented beverages have recently gained consumers’ interest, particularly due to their intrinsic functional properties and presence of beneficial microorganisms. Three variants containing 5%, 10%, and 15% (*w*/*w*) of sweet blue lupin (*Lupinus angustifolius* L. cv. “Boregine”) seeds were inoculated with kefir grains and incubated at 25 °C for 24 h. After processing, beverages were stored in refrigerated conditions (6 °C) for 21 days. Changes in microbial population, pH, bioactive compounds (polyphenolics, flavonoids, ascorbic acid), reducing sugars, and free amino acids were estimated. Additionally, viscosity, firmness, color, and free radicals scavenging properties were determined. Results showed that lactic acid bacteria as well as yeast were capable of growing well in the lupin matrix without any supplementation. During the process of refrigeration, the viability of the microorganisms was over the recommended minimum level for kefir products. Hydrolysis of polysaccharides as well as increase of free amino acids was observed. As a result of fermentation, the beverages showed excellent DPPH, ABTS^+·^, ^·^OH, and O_2_^−^ radicals scavenging activities with a potential when considering diseases associated with oxidative stress. This beverages could be used as a new, non-dairy vehicle for beneficial microflora consumption, especially by vegans and lactose-intolerant consumers.

## 1. Introduction

Increasing consumers’ demand for healthy foods and awareness of the impact of dietary habits in human well-being has prompted the efforts of developing novel foods with defined health benefits [1,2,3,4,5]. Nowadays, a great number of novel functional foods are available on the market with dairy foods and beverages representing an important segment [1]. One of the most popular dairy foods with undisputed benefits from consumption is kefir, that can be prepared from different milk types, such as goat, buffalo, sheep, camel, or cow via microbial fermentation (inoculating milk with kefir grains, consisting of lactic acid bacteria and yeasts) [6,7]. In the last decades, many scientific reports highlighted nutritional properties of kefir and health benefits owing to its antimicrobial, anticancer, gastrointestinal tract effects, gut microbiota modulation, cholesterol-lowering activity, and anti-diabetic effects [3,6,7,8,9]. Recently, milk consumption has been declining, and consumer demand for cow’s milk alternatives increased as a result of the increase in the diagnosis of lactose intolerance, allergies, and cholesterol issues [10,11,12,13]. Furthermore, the ongoing trend of vegetarianism, with an increasing number of vegan/vegetarian consumers, has established a massive worldwide importance of non-dairy plant-based dairy substitutes [10,14,15,16]. Not wishing to consume food of animal origin, vegan consumers are looking for substitutes that could enrich their diet and contribute to a good health. Moreover, plant-based milks and beverages play a pivotal role in meeting this demand as substitutes for bovine milk in areas where it is expensive, or in consumption by infants and adults who are allergic to animal milk [15,16]. An alternative way of producing kefir is to utilize non-dairy substrates such as fruits and molasses to produce sugary kefir [6]. The new kefir-like products might represent important foods providing live microorganisms to vegetarians with a limited availability of fermented products, and some interesting results have been reported for utilization of plant substrates such as flaxseed oil cake [11], rice [17], nuts [18,19], and fruits and vegetables [20,21,22]. In developing countries, the ever-growing population and the malnutrition problem caused by protein deficits necessitate an increased supply for food proteins [23,24]. Food of animal origin has the disadvantage of being expensive to produce because of the biological inefficiency of converting plant proteins into animal proteins [24]. In developing countries, there is awareness of the increasing environmental burden [23]. Therefore, the industrial exploitation of new protein sources such as plant proteins to broaden the range and variety of foods is becoming an inevitable trend [25,26,27]. Moreover, World Health Organization advises frequent consumption of vegetable foods instead of animal foods with considerable amount of saturated fats and cholesterol [28].

Lupin is a grain crop with excellent nutritional value and stands out for its high protein content [28,29,30,31]. Moreover, it has both health and commercial value in the food industry, and is rich in phytochemicals (such as polyphenols, phytosterols, squalene) [25,28,29,32,33]. About 400 species of lupin (genus: *Lupinus* L.) have been found in nature. Among them, only few species, white lupin (*L. albus* L.), blue lupin (*L. angustifolius* L.), yellow lupin (*L. luteus* L.), and pearl or Tarrwi lupin (*L. mutabilis* Brit. Fl. Gard. [Sweet] Ser.) have been extensively studied for their agronomical characteristics and nutritional values [32]. Being a legume, lupin protein is a vegetable protein that has similar attributes to soybean protein, and it could be an alternative to soybean in the food industry. Besides, lupin does not contain gluten, thus it could be used as a functional ingredient in gluten-free foods [27,34,35]. The industrial shift of lupin seed utilization from feed to food has recently increased the scientific interest to explore its phytochemical composition and biological activities. Lupin products are valued for their GMO (genetically modified organism) free status, functional food properties, nutritional and health benefits, and seem to be promising as a source of an innovative food ingredient for the food industry [25,26,36]. However, contrary to what some people believe, lupin seed is not a novel food, as it has been used for this purpose in Mediterranean, African and Andean regions for many centuries [4,24,29]. The current use of lupin whole seed as a food is limited to local dietary habits, and lupin products are mainly produced for people with special dietary needs: Athlete nourishment and various products dedicated to celiac individuals, vegans, and vegetarians [4,36]. Lupin seeds are often consumed as an appetizer, furthermore they can be milled into flour and used for the manufacturing of baked goods and pastry products [31,35]. Moreover, lupin could be used for production dairy additives and dairy substitutes such as plant milk and yogurt [16,33,34,35,37,38]. Fermentation of lupin was successfully carried out with the use of moulds and yeast (*Aspergillus* sp., *Candida* sp., *Rhizopus oryzae, Saccharomyces* sp.), bacteria (*Bacillus subtilis*, *Bifidobacterium* sp., *Lactobacillus* sp., *Pediococcus* sp.) or spontaneously [27,39,40,41,42,43]. There are no studies available concerning lupin seeds fermented by kefir grains.

No studies have been published on the use of blue lupin (*L. angustifolius*) seeds for development of kefir-like fermented semi-solid beverages. We hypothesized that fermentation of lupin seeds with kefir grains will allow to obtain new products with high added values in comparison with unprocessed seeds. Thus, the aim of the presented study is to produce beverage based on various concentrations of lupin fermented by kefir grains and evaluation of its bioactivity, microbiological, and physiochemical properties during refrigerated storage for 21 days.

## 2. Results and Discussion

### 2.1. The Lactic Acid Bacteria (LAB) and Yeast Survivability during Cold Storage

LAB (lactic acid bacteria) and yeast concentration after fermentation, and viability over storage period in the beverage formulations (A—sample with 5% (*w*/*w*) lupin seeds content, B—sample with 10% (*w*/*w*) lupin seeds content, C—sample with 15% (*w*/*w*) lupin seeds content) are presented in Table 1. At any time, the bacterial and yeast counts were maintained in the samples over the recommended for kefir level >10^7^ CFU/g and >10^4^ CFU/g, for bacteria and yeast, respectively. However, during storage, some significant fluctuations (*p* < 0.05) of both LAB and yeast counts were observed, which may be linked with different nutrients availability in the samples. High survivability of kefir microflora in plant-based beverages was also reported [11,18,19]. Jimenéz-Martínez et al. observed high LAB survivability in lupin milk [16], whereas Schlegel et al. noted comparable survivability of different *Lactobacillus* strains in fermented lupin protein isolate [43]. However, values reported by Zaworska et al. for fermented lupin seeds [40], are lower than reported in present study. On day 21 significant differences between bacterial (*p* < 0.05) as well as fungal (*p* < 0.05) counts were observed. The fact that microorganisms in the samples remained highly concentrated might be due to low (6 °C) storage temperature, as it widely known that refrigerated conditions are one of the pivotal points maintaining LAB and yeast viability in fermented beverages, increasing their shelf-life [1,11,12].

### 2.2. The Changes of pH, Reducing Sugars and Free Amino Acids Level

**Figure 1:**
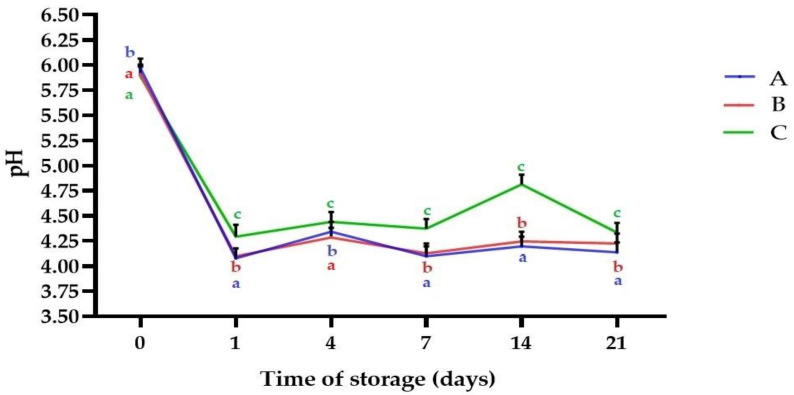
pH of the samples during storage time. Means with different lowercase are significantly different at *p* < 0.05. **A**—sample with 5% (*w*/*w*) lupin seeds content, **B**—sample with 10% (*w*/*w*) lupin seeds content, **C**—sample with 15% (*w*/*w*) lupin seeds content.

**Figure 2:**
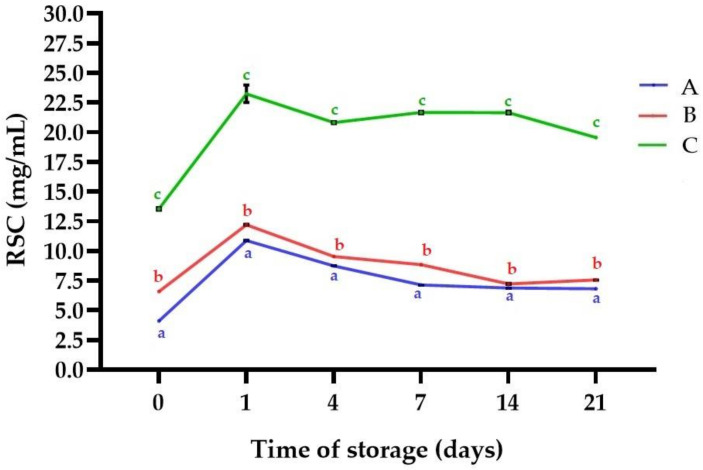
Reducing sugars content (RSC) of the samples during storage time. Means with different lowercase are significantly different at *p* < 0.05. **A**—sample with 5% (*w*/*w*) lupin seeds content, **B**—sample with 10% (*w*/*w*) lupin seeds content, **C**—sample with 15% (*w*/*w*) lupin seeds content.

**Figure 3:**
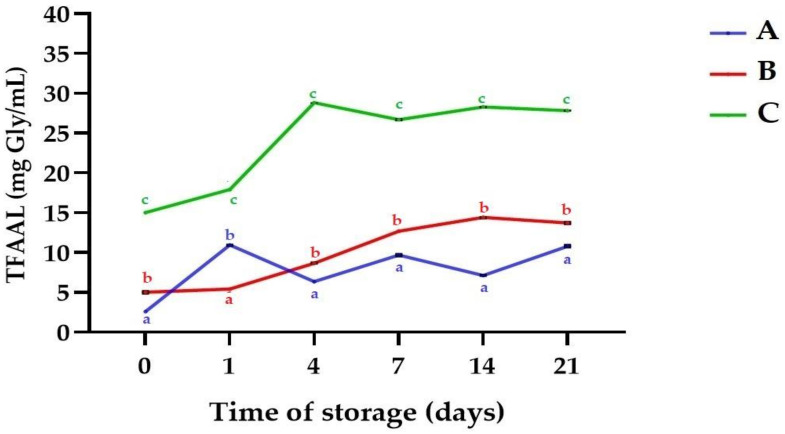
Total free amino acids level (TFAAL) of the samples during storage time. Means with different lowercase are significantly different at *p* < 0.05. **A**—sample with 5% (*w*/*w*) lupin seeds content, **B**—sample with 10% (*w*/*w*) lupin seeds content, **C**—sample with 15% (*w*/*w*) lupin seeds content.

Figure 1 shows the results obtained for pH profile of the samples plotted against time. A significant decrease of pH to the range 4.09 ± 0.01 (A)–4.31 ± 0.02 as a result of fermentation was observed (*p* < 0.05). Those values are comparable to results reported by Camacho et al. for *L. albus* fermented with various *Lactobacillus* strains [41], but higher than reported by Klupsaite et al. for *L. angustifolius* fermented with *Pediococcus pentosaceus* KTU05-9 [27]. The observed decrease of pH is also in line with findings of other authors for plant matrices fermented with kefir grains such as hazelnut milk [18], and flaxseed oil cake [11]. However, slight deacidification was noticed during cold storage time (*p* < 0.05). The acidity of fermented products is commonly maintained or decreased during storage, a fact that is linked with microbial activity, which is a continuous fermentation process in which LAB and yeast assimilate carbohydrates [11,44]. It was reported that some of the common problems in legume-based fermented foods is acidity, which during the fermentation of kefir is of great importance, because of inhibitory effects against spoilage and pathogenic microorganisms [41,42]. The pH level was observed to be below 4.7, which is considered essential maximum for microbial stability [16]. In the present study we hypothesized that kefir microflora would use some structural sugars. Indeed, as presented in Figure 2 there was a significant increase in reducing sugars content (RSC) after fermentation in comparison to the non-fermented samples (*p* < 0.05). After fermentation, the highest RSC was noticed for sample C (23.24 ± 0.74 mg/mL). The increased RSC may be attributed with enzymatic hydrolysis of oligo-, and polysaccharides from lupin seeds. Plant matrices are rich in non-starch polysaccharides, i.e., arabinoxylans, β-glucans, cellulose, and lignin, and often exist in composite structures with other small molecular weight compounds, e.g., phenolics, flavonoids, and minerals. During fermentation LAB and yeast produce different types of hydrolases, which partially or fully degrade the oligosaccharides into simple sugars [40]. The enzymatic activity is responsible for biopolymers degradation, leading to cell wall degradation (softening). LAB make the food easily digestible, decreasing the level of high-chain carbohydrates and some indigestible poly- and oligosaccharides. This is of great importance from the sensorial, physicochemical and dietary point of view, because carbohydrates digestibility is related to many human health issues [40,45]. The hydrolysis of oligosaccharides and production of simple sugars was reported by Camacho et al., who fermented lupin seeds with *L. acidophilus* B-1910 and *L. fermentum* B-585 [41]. Also, Romero-Espinoza et al. observed that probiotic bacteria and yeasts partially degraded oligosaccharides of fermented *L. mutabilis* [46]. However, in present study a decreased trend of RSC was generally observed in the following days of cold storage (*p* < 0.05). This behavior is linked with the utilization of produced sugars, required for maintaining of microbial metabolic activity, and was reported in other studies [11]. As presented in Figure 3, total free amino acids level (TFAAL) of all the samples significantly increased as a result of fermentation and an increase trend was observed during cold storage, which indicates directly progressive proteolysis (*p* < 0.05). On day 21 TFAAL in a range 10.79 ± 0.09 mg Gly/mL (sample A)–27.81 ± 0.03 mg Gly/mL (sample C) was noticed. It is known, that upon fermentation microbial proteases are released and degrade to a certain extend the proteins in a composite food matrix [45]. Many biochemical transformations which alter the ratio of nutritional compounds occur during the fermentation process, and these changes affect the characteristics of processed foods such as digestibility and amino acid profile [25,47,48]. The increased TFAAL level is in line with findings of Bartkiene et al., who showed that lupin protein digestibility and amino acids profile can be improved using lactofermentation [26], and also with conclusions of Kasprowicz-Potocka et al., who reported that fermentation of lupin seeds using yeast increased the concentration of essential amino acids [39].

### 2.3. The Changes of Total Polyphenolics, Flavonoids, and Ascorbic Acid Contents

The changes of total polyphenolics content (TPC), total flavonoids content (TFC) and ascorbic acid content (AAC) are summarized in Table 2. As expected, the content of bioactive compounds was modified by the fermentation process. A significant increase of TPC (almost 2-fold) in comparison to un-fermented samples was noticed for all the samples on day 21 (*p* < 0.05). Similarly, a significant increase of TFC was found in all samples (*p* < 0.05). The highest TPC (35.86 ± 0.03 mg GAE/mL) and TFC (33.89 ± 0.08 QE/mL) was observed in sample C on day 21. Siger et al. found that *L. angustifolius* seeds are a rich source of polyphenolics and flavonoids with high antioxidant potential [49]. Moreover, these results correspond well with those obtained by Bartkiene et al. who reported higher TPC and TFC content in fermented lupin [25]. Generally, the major phenolic compounds identified in lupin species belong to subclass flavones, phenolic acids, and isoflavones [32,49,50,51,52]. The main identified flavones in the group are aglycone and/or glycosides of luteolin, apigenin, and diosmetin, while the principal contribution in the isoflavone group is from genistein and its derivatives. In phenolic acids, protocatechuic acid (dihydroxybenzoic acid) and *p*-hydroxybenzoic acid are the major representative components. Although flavones are found in higher quantities, many of the isoflavones present in lupin species get more importance because of their nonsteroidal phytoestrogenic activity in mammals [32,49]. The effect of fermentation on the TPC and TFC (attributed to delinking of some phenolic compounds that were bounded to proteins and cell wall carbohydrates) and antioxidant activity of plant matrices has been reported in numerous studies [11,12,14,25]. It is of particular importance, because phenolic compounds need to be in a soluble form to enter the human blood circulation system and bring about their antioxidant properties [45]. Generally, fermentation did not affect ascorbic acid level, only on day 21 in sample C, a significantly higher AAC was found (*p* < 0.05). A similar effect was reported for flaxseed oil cake kefir-like beverages [11].

### 2.4. The Changes of Reducing Power and Radical Scavenging Activties

The conducted research proved that the reducing power, DPPH, ABTS^+·^, ^·^OH and O_2_^−^ radicals scavenging activities were effectively increased as a result of fermentation (*p* < 0.05), what is summarized in Table 3. It is noteworthy, that fermented beverages did not lose their antioxidative properties as a consequence of cold storage, but, in fact, exhibited increased antioxidative activity. The highest DPPH (94.27 ± 0.06%), and ABTS^+·^ (92.37 ± 0.11%) inhibition was noticed for sample C on day 21, whereas for this sample the highest ·OH scavenging activity was found on day 14. However, the highest O_2_^−^ scavenging activity (95.47 ± 0.08%) was noticed for sample B on day 21. Lupin is reported to possess the antioxidant activity, and of all the bioactive compounds present in lupin seeds, phenolic compounds are primarily responsible for the antioxidant capacity of the seeds [29,32,49]. Bartkiene et al. also reported higher antioxidant activity of fermented lupin seeds wholemeal and protein isolates when compared with non-fermented ones [25]. Thambiraj et al. found that polysaccharide and mono-sugar fractions from *L. angustifolius* seeds have strong ABTS^+·^ scavenging activity [53]. Liu et al. observed a greater reducing power of milk-kefir and soymilk-kefir than that of respective milks from which they were made [54]. Similarly, peanut milk kefir extract displayed stronger antioxidant properties than peanut milk alone, suggesting a fermentation impact of kefir grain on peanut milk’s efficacy [6]. Generally, the increase of antioxidant activity of the fermented products is linked with microbial activity, production and liberation of certain bioactive compounds which demonstrate reducing power and react with free radicals to stabilize and terminate radical chain reactions [11,14]. Presence of excessive free radicals in a biological system can lead to the process of DNA (deoxyribonucleic acid) damage and hence can cause serious diseases [53]. Oxidative stress is associated with the development of so-called “civilization diseases”, such as cancer, stroke, myocardial infarction, inflammation, as well as the degenerative processes associated with aging [55]. Hence, radical scavenging activity is an essential process by which oxidative free radicals can be removed and DNA damage can be prevented, and consumption of foods rich in antioxidants plays an essential role in the prevention of these diseases [53]. Thus, it is reasonable to conclude that fermentation of lupin with kefir grains can result in new compounds with health-modulating potential.

### 2.5. The Changes of Color

Table 4 presents the color parameters of both fermented and non-fermented samples. As can be seen, fermentation significantly increased lightness (L*) values (*p* < 0.05). A similar increase of L* as a result of fermentation was observed for flaxseed oil cake kefir-like beverage. It was observed that yellowness (b*) values of the samples decreased (*p* < 0.05). An increase of a* value (redness) of sample A was found, whereas values of samples B and C decreased (*p* < 0.05). A significant fluctuations of color parameters of fermented samples were observed during the storage time (*p* < 0.05). Those changes can be attributed to pH variations, and oxidation of some pigments presented in the raw material [11]. On the contrary, Jimenéz-Martínez et al. observed lower a* and higher b* values for lupin-based plant milk [16].

### 2.6. Viscosity and Textural Changes

At the beginning, the viscosity of the samples showed significant differences because of lupin seeds concentration as presented in Table 5 (*p* < 0.05). It was observed that after fermentation and up to day 4 the viscosity of the samples increased (*p* < 0.05). Similarly, the viscosity of flaxseed oil cake kefir-like beverages increased, which was linked with production of polysaccharide kefiran [11]. However, since day 7 a decrease of the samples’ viscosity was found (*p* < 0.05). Likewise, the firmness values of the beverages showed similar trend. This may be linked with polysaccharides hydrolysis, leading to cell wall degradation (indicated by formation of RSC), and, finally softening of the beverages’ matrix. Additionally, proteins play a significant role in textural characteristics formation due to proteins gelation properties. In this context, the changes in the texture and viscosity of the samples can be also explained by the constant breakdown of the proteins bonds as a result of proteolysis and formation of TFAAL [14,37].

## 3. Materials and Methods

### 3.1. Materials and Reagents

Seeds of sweet blue lupin (*Lupinus angustifolius* L. cv. “Boregine”—with low alkaloid content <0.01%) were kindly donated by Saatzucht Steinach GmbH & Co KG (Steinach, Germany). Commercial kefir grains (Yoghurt-Tek^®^, Lactoferm Kefir Series, Kefir-31, consisting of *Lactococcus lactis* subsp. *cremoris, Lactococcus lactis* subsp. *lactis* biovar *diacetylactis*, *Leuconostoc mesenteroides* subsp. *cremoris*, *Lactobacillus delbrueckii* subsp. *bulgaricus*, and *Saccharomyces cerevisiae*) were obtained from Biochem s.r.l. (Rome, Italy). Sodium hydroxide, hydrogen peroxide, disodium phosphate, monosodium phosphate, 2,2-diphenyl-1-picrylhydrazyl (DPPH), 2,2′-azino-bis(3-ethylbenzothiazoline-6-sulfonic acid) (ABTS), methanol, Folin–Ciocalteu’s reagent, sodium carbonate, sodium chloride, gallic acid, sodium nitrite, aluminum chloride, quercetin, 3,5-dinitrosalicylic acid, sodium tartrate tetrahydrate, acetic acid, sodium acetate, potassium ferricynide, trichloroacetic acid, oxalic acid, 2,6-dichlorophenolindophenol, ferric chloride, ninhydrin, glacial acetic acid, cadmium chloride, tris(hydroxymethyl)aminomethane, pyrogallol, ortophenantroline, glycine were purchased from Sigma Aldrich (Sigma Aldrich, Darmstadt, Germany). Glucose, hydrochloric acid and ammonium thiocyanate were supplied from Chempur (Chempur, Piekary Śląskie, Poland). All reagents were of analytical grade. MRS (de Man, Rogosa and Sharpe) agar, and Sabouraud agar with chloramphenicol were obtained from Merck (Merck, Darmstadt, Germany).

### 3.2. Samples Preparation and Fermentation

The process of lupin-based beverages preparation consisted of few steps. Firstly, lupin seeds were mixed with distilled water (*w*/*w*) to obtain three variants: A (5%), B (10%), C (15%), and incubated in refrigerator (6 °C) for 24 h. Then, the aqueous phase was replaced with fresh sterile distilled water, and the mixtures were heated at 90 °C for 20 min with constant stirring (250 rpm). Then the mixtures were cooled down to room temperature. The samples were homogenized for 5 min with a homogenizer (SilentCrusherM, Heldolph, Germany) at 12,000 rpm. After homogenization the mixtures were dispensed into containers and were pasteurized by heating for 30 min at 60 °C, then cooled down and stored in a refrigerator day before the fermentation. Kefir-like beverages were produced by mixing 500 mL of a particular variant (pre-heated to 25 °C) with 5 g of kefir grains (containing 1.6 × 10^7^ ± 0.40 CFU/g of LAB and 1.5 × 10^7^ ± 0.11 CFU/g of yeast) and fermented at 25 °C in 100 mL closed sterile plastic containers for 24 h. After processing, beverages were cooled down and stored at 6 °C for 21 days. The analyses were performed after 1, 4, 7, 14, and 21 days of storage. The non-fermented reference samples were treated the same way but without kefir grains addition, which served for comparison.

### 3.3. Microbiological Analyses and pH Determination

During the overall storage, samples (10 g) were collected and diluted with 90 mL of sterile physiological saline (0.9% NaCl), and serial dilutions were prepared [11]. Lactic acid bacteria counts were determined on MRS medium (Merck, Darmstad, Germany) after incubation at 37 °C under anaerobic conditions for 72 h, whereas yeast counts were determined on Sabouraud Agar supplemented with chloramphenicol at 25 °C for 72 h. The enumeration of microorganisms was performed in triplicate and the viable cell counts were expressed as CFU/g of the samples. The pH of non-fermented and fermented samples were measured directly at 25 °C using a pH-meter (CP-411, Elmetron, Zabrze, Poland).

### 3.4. Supernatants Preparation

To obtain clear fluids for analyses the samples were prepared as described elsewhere [11]. Briefly, the samples were transferred into 1.5-mL Eppendorf tubes and centrifuged at 14,000 rpm/min for 10 min at 20 °C (Centrifuge 5418 Eppendorf, Warsaw, Poland). The supernatants of the particular type of sample were mixed and filtered through 0.22-µm nylon membrane filters (Sigma-Aldrich, Darmstadt, Germany). The obtained clear fluids were used for further analyses.

### 3.5. Determination of Total Polyphenolic Content (TPC), Total Flavonoid Content (TFC), Reducing Sugars Content (RCS), Total Free Amino Acids Level (TFAAL), and Ascorbic Acid Content (AAC)

The total polyphenolics content of each supernatant was determined by Folin–Ciocalteu method [11]. The supernatants (100 µL) were mixed with 6 mL of distilled water and 0.5 mL of Folin–Ciocalteu’s reagent. After 3 min, 1.5 mL of saturated Na_2_CO_3_ solution was added and the mixture was incubated for 30 min in darkness at 40 °C. The absorbance of the mixture was measured at 765 nm (UV-Vis Thermo Scientific Evolution 220 spectrophotometer). The concentration of TPC was calculated as mg of gallic acid equivalents (GAE) per mL of sample (mg GAE/mL).

The total flavonoids content (TFC) of each sample was determined by mixing 250 µL of supernatant with 1 mL of distilled water and 75 µL of 5% NaNO_2_ solution. After 5 min, 75 µL of 10% AlCl_3_ solution was added, and the mixture was allowed to stand for 6 min before the addition of 250 µL of 1 M NaOH. The total volume mixture was made up to 3 mL with distilled water, and then the absorbance was measured at 510 nm (UV-Vis Thermo Scientific Evolution 220 spectrophotometer). Quercetin was used for a calibration curve, and the results were expressed as mg of quercetin equivalents (QE) per mL of the sample (mg QE/mL) [11].

The reducing sugars content (RSC) was determined by DNS (3,5-dinitrosalicylic acid) method. A total of 10 g of DNS was dissolved in 200 mL of distilled water by continuous stirring, then slowly 16 g of NaOH (dissolved first in 150 mL of distilled H_2_O) was added. The mixture was incubated at 50 °C with stirring to obtain a clear solution. Then 403 g of potassium sodium tartrate tetrahydrate was added in small portions. The mixture was filtered using a paper filter and the volume was made up to 1000 mL with distilled water. One milliliter of supernatant was mixed with 1 mL of 0.05 m acetate buffer (pH 4.8), and 3 mL of DNS reagent was added, then vigorously shaken. The mixtures were incubated in boiled water for 5 min then cooled at room temperature. The absorbance values were then recorded at 540 nm (UV-Vis Thermo Scientific Evolution 220 spectrophotometer). Glucose in acetate buffer was used for a calibration curve [11].

Total free amino acids level (TFAAL) was determined as described elsewhere with a slight modification [56]. A quantity of 1 mL of the supernatants were mixed with 2 mL of a Cd-ninhydrin reagent (0.8 g ninhydrin was dissolved in a mixture of 80 mL ethanol and 10 mL glacial acetic acid, followed by the addition of 1 g CdCl_2_ dissolved in 1 mL of distilled water). The mixtures were vortexed and heated at 84 °C for 5 min and cooled in ice-water, and the absorbance was determined at 507 nm. The results were expressed as milligram Gly per mL of the sample by reference to a standard curve which was first prepared using glycine at various concentrations.

The Tillmans titration method involving a reduction of 2,6-dichlorophenolindophenol was used to determine the ascorbic acid content [57]. Two milliliters of supernatant were mixed with 2 mL of oxalic acid solution (2%) and vigorously shaken. The solution was quickly titrated with 2,6-dichlorophenolindophenol until pink color held for 30 s. The content of ascorbic acid was expressed as milligrams per mL of the sample.

### 3.6. Determination of Reducing Power and Radical Scavenging Activity

To determine the reducing power, the supernatants (500 μL) were placed in a tube, to which 1.25 mL of phosphate buffer solution (0.2 M, pH 6.6), as well as 1.25 mL of 1% potassium ferricyanide solution were added. After incubation at 50 °C for 20 min, 1.25 mL of trichloroacetic acid solution were added to the tube. Next, 1.25 mL of supernatant obtained by centrifugation at 3000 rpm for 10 min was diluted with 1.25 mL of deionized water. Finally, 0.25 mL of 0.1% ferric chloride solution was added to complete the assay. The absorbance was determined at 700 nm which represented the reducing power [14].

DPPH, ABTS^+·^, ^·^OH, and O_2_^−^ radicals scavenging activities were determined according to the procedures as described in previous study [14]. In brief, the DPPH radical scavenging activity was determined by mixing 1 mL of the supernatants with 1 mL of 0.01 mM DPPH methanolic solution. The absorbance was measured at 517 nm. Three mL of ABTS^+·^ solution were mixed with 50 µL of the supernatants and the absorbance was measured at 734 nm. To determine ^·^OH radical scavenging activity, 1 mL of supernatants and 1.5 mL of ortophenantroline solution (0.005 mmol/L) were mixed with 2 mL of phosphate buffer (pH 7.4, 0.05 mol/L). Then 1 mL of FeSO_4_ solution (0.0075 mol/L) was added and then mixed with 1 mL of H_2_O_2_ (0.1%), and finally supplemented with distilled water to a total volume of 10 mL. The reaction solution were kept at 37 °C for 1 h in darkness, then the absorbance was measured at 536 nm. To determine O_2_^-^ radical scavenging activity, 3 mL of 50 mmol/L (pH 8.2) Tris-HCl buffer were mixed with 1 mL of the supernatants. These mixtures were mixed with a pyrogallol solution (0.3 mL, 7 mmol/L, preheated to 25 °C) and allowed to react for exactly 4 min, then 1 mL od 10 mmol/L of HCl was added to terminate the reaction, and absorbance was measured at 318 nm.

### 3.7. Texture Profile Analysis and Viscosity Measurements

Texture profiles were performed at room temperature using a Zwick/Roell 2,5 Z equipment (Zwick/Roell, Ulm, Germany), equipped with a cylindrical probe (diameter 40 mm). The samples were analyzed directly, penetration rate into the samples was 10 mm/s, and the penetration depth was 25 mm. From the results of the force-time curves, the firmness and hardness were calculated. The viscosity measurements were performed in a rheometer (AR G2, TA Instruments Ltd., New Castle, DE, USA). The samples were analyzed at 20 °C using a stainless steel cone plate having a diameter of 62 mm. Steady-state flow measurements were carried out at a shear rate 50 s^−1^ and the viscosity values were obtained from the TA Rheology Advantage Data Analysis equipment software V 5.4.7. (TA Instruments, New Castle, DE, USA).

### 3.8. Statistical Analysis

All data were expressed as mean ± standard deviation (SD). Statistical significance was determined using an analysis of variance (two-way ANOVA) followed by NIR Fisher test. The values were considered as significantly different when *p* < 0.05. All analyses were performed with Statistica version 10 (StatSoft Polska, Kraków, Poland).

## 4. Conclusions

The findings of this work indicated that blue lupin seeds can be fermented with kefir grains, resulting in a novel functional beverage type, with high beneficial microflora (lactic acid bacteria and yeast) viability. The development of kefir-fermented product is possible, allowing the consumption of beneficial microorganisms by consumers where non-dairy alternatives are desired. The developed products are a rich source of bioactive compounds (such as polyphenolics, flavonoids, amino acids) with significant radical scavenging activities. It should be noted that consumption of the beverages may be potentially beneficial to the human organism, especially when considering diseases associated with oxidative stress. However, it is still needed to conduct more research focusing on the in vivo analysis of the benefits of consuming these products and the impact on the functioning of the body.

## Figures and Tables

**Table 1:** Lactic acid bacteria (LAB) and fungi counts during storage time.

	Time of Storage (Days)				
	1	4	7	14	21
LAB CFU/g					
A	1.47 × 10^9^ ± 0.18 ^Ac^	7.05 × 10^7^ ± 1.86 ^Ba^	1.53 × 10^8^ ± 0.08 ^Ca^	1.92 × 10^7^ ± 0.10 ^Da^	4.18 × 10^8^ ± 0.39 ^Ea^
B	2.86 × 10^8^ ± 3.02 ^Ab^	1.25 × 10^9^ ± 0.26 ^Ba^	7.54 × 10^7^ ± 1.92 ^Cb^	3.25 × 10^7^ ± 0.41 ^Da^	7.45 × 10^8^ ± 2.19 ^Db^
C	1.82 × 10^7^ ± 0.00 ^Aa^	1.53 × 10^8^ ± 0.30 ^Bb^	1.13 × 10^8^ ± 0.19 ^Cab^	1.03 × 10^9^ ± 0.38 ^Db^	5.18 × 10^8^ ± 5.76 ^Dc^
Yeast CFU/g					
A	1.47 × 10^9^ ± 0.18 ^Aa^	3.36 × 10^7^ ± 1.29 ^Ba^	3.45 × 10^7^ ± 0.07 ^Ca^	5.87 × 10^6^ ± 7.90 ^Da^	1.26 × 10^7^ ± 0.06 ^Ea^
B	1.68 × 10^9^ ± 0.02 ^Aa^	8.59 × 10^7^ ± 0.06 ^Bb^	1.20 × 10^8^ ± 0.22 ^Cb^	1.40 × 10^7^ ± 0.19 ^Db^	6.23 × 10^7^ ± 1.61 ^Eab^
C	3.15 × 10^9^ ± 3.32 ^Ab^	9.49 × 10^7^ ± 0.45 ^Bc^	2.25 × 10^8^ ± 0.07 ^Cc^	1.38 × 10^8^ ± 0.18 ^Dc^	8.41 × 10^7^ ± 1.35 ^Eb^

**A**—sample with 5% (*w*/*w*) lupin seeds content, **B**—sample with 10% (*w*/*w*) lupin seeds content, **C**—sample with 15% (*w*/*w*) lupin seeds content. Values are means ± standard deviation of triplicate determinations. Means with different lowercase in the same column are significantly different at *p* < 0.05. Means with different uppercase in the same raw are significantly different at *p* < 0.05.

**Table 2:** Total polyphenolics content (TPC), total flavonoids content (TFC), and ascorbic acid content (AAC) changes of the samples during storage.

	Time of Storage (Days)					
	Unfermented	1	4	7	14	21
TPC (mg GAE/mL)						
A	10.32 ± 0.05 ^Aa^	11.60 ± 0.03 ^Ba^	12.71 ± 0.03 ^Ca^	16.85 ± 0.03 ^Da^	18.81 ± 0.31 ^Ea^	19.32 ± 0.03 ^Fa^
B	15.95 ± 0.03 ^Ab^	16.45 ± 0.00 ^Bb^	20.35 ± 0.00 ^Cb^	24.87 ± 0.07 ^Db^	27.26 ± 0.03 ^Eb^	30.54 ± 0.08 ^Fb^
C	17.93 ± 0.08 ^Ac^	18.94 ± 0.00 ^Bc^	22.28 ± 0.14 ^Cc^	25.86 ± 0.03 ^Dc^	28.71 ± 0.04 ^Ec^	35.86 ± 0.03 ^Fc^
TFC (mg QE/mL)						
A	13.75 ± 0.07 ^Aa^	15.13 ± 0.04 ^Ba^	18.47 ± 0.04 ^Ca^	20.30 ± 0.05 ^Da^	22.54 ± 0.04 ^Ea^	23.92 ± 0.04 ^Fa^
B	21.27 ± 0.04 ^Ab^	22.17 ± 0.04 ^Bb^	23.42 ± 0.04 ^Cb^	27.75 ± 0.05 ^Db^	28.29 ± 0.05 ^Eb^	33.42 ± 0.04 ^Fb^
C	23.90 ± 0.10 ^Ac^	31.54 ± 0.07 ^Bc^	31.17 ± 0.05 ^Cc^	31.48 ± 0.14 ^Dc^	32.60 ± 0.08 ^Ec^	33.89 ± 0.08 ^Fc^
AAC (mg/mL)						
A	0.05 ± 0.01 ^ABa^	0.05 ± 0.00 ^ABa^	0.04 ± 0.01 ^Aa^	0.04 ± 0.01 ^Aa^	0.05 ± 0.01 ^ABa^	0.06 ± 0.01 ^BCa^
B	0.06 ± 0.01 ^Aab^	0.04 ± 0.01 ^ABa^	0.05 ± 0.02 ^ABa^	0.06 ± 0.01 ^ABa^	0.06 ± 0.01 ^Aab^	0.06 ± 0.01 ^Aa^
C	0.07 ± 0.00 ^Ab^	0.05 ± 0.01 ^Bb^	0.07 ± 0.00 ^Ab^	0.07 ± 0.01 ^Ab^	0.07 ± 0.00 ^Ab^	0.11 ± 0.01 ^Cb^

**A**—sample with 5% (*w*/*w*) lupin seeds content, **B**—sample with 10% (*w*/*w*) lupin seeds content, **C**—sample with 15% (*w*/*w*) lupin seeds content. Values are means ± standard deviation of triplicate determinations. Means with different lowercase in the same column are significantly different at *p* < 0.05. Means with different uppercase in the same raw are significantly different at *p* < 0.05.

**Table 3:** Reducig power (RP), 2,2-diphenyl-1-picrylhydrazyl (DPPH), 2,2′-azino-bis(3-ethylbenzothiazoline-6-sulfonic acid) (ABTS), O_2_^−^, and ^·^OH radical scavenging activity of fermented beverages and unfermented (control) samples.

	Time of Storage (Days)					
	Unfermented	1	4	7	14	21
	RP (700 nm)					
A	0.449 ± 0.01 ^Aa^	0.630 ± 0.02 ^Bb^	0.635 ± 0.02 ^Cb^	0.422 ± 0.05 ^Da^	0.628 ± 0.02 ^Ba^	0.600 ± 0.01 ^Ea^
B	0.513 ± 0.02 ^Ab^	0.593 ± 0.02 ^Ba^	0.587 ± 0.01 ^Ca^	0.753 ± 0.01 ^Db^	0.732 ± 0.01 ^Eb^	0.754 ± 0.01 ^Fb^
C	0.621 ± 0.01 ^Ac^	0.643 ± 0.01 ^Bc^	0.681 ± 0.01 ^Cc^	0.782 ± 0.01 ^Dc^	0.825 ± 0.01 ^Ec^	0.754 ± 0.01 ^Fb^
	DPPH inhibition (%)					
A	65.50 ± 0.27 ^Aa^	67.98 ± 0.22 ^Ba^	69.79 ± 0.00 ^Ca^	85.54 ± 0.89 ^Da^	87.91 ± 0.36 ^Ea^	91.48 ± 0.06 ^Fa^
B	65.32 ± 0.06 ^Aa^	87.27 ± 0.07 ^Bb^	90.35 ± 0.23 ^Cb^	90.10 ± 0.15 ^Db^	90.74 ± 0.00 ^Eb^	92.90 ± 0.27 ^Fb^
C	75.06 ± 0.57 ^Ab^	88.63 ± 0.06 ^Bc^	90.79 ± 0.00 ^Cb^	91.99 ± 0.12 ^Dc^	93.06 ± 0.00 ^Ec^	94.27 ± 0.06 ^Fc^
	ABTS inhibition (%)					
A	42.60 ± 0.06 ^Aa^	49.53 ± 0.37 ^Ba^	57.76 ± 0.31 ^Ca^	64.77 ± 0.39 ^Da^	70.86 ± 0.34 ^Ea^	82.05 ± 0.40 ^Fa^
B	44.06 ± 0.14 ^Ab^	60.23 ± 0.33 ^Bb^	61.57 ± 0.13 ^Cb^	72.40 ± 0.13 ^Db^	78.23 ± 0.05 ^Eb^	86.35 ± 0.07 ^Fb^
C	47.41 ± 0.10 ^Ac^	69.28 ± 0.27 ^Bc^	73.88 ± 0.49 ^Cc^	79.34 ± 0.21 ^Dc^	88.72 ± 0.07 ^Ec^	92.37 ± 0.11 ^Fc^
	O_2_^−^ inhibition (%)					
A	32.49 ± 0.09 ^Aa^	66.67 ± 0.00 ^Ba^	66.70± 0.03 ^Ba^	70.32 ± 0.00 ^Ca^	85.71 ± 0.00 ^Ca^	87.62 ± 0.00 ^Da^
B	44.29 ± 0.05 ^Ab^	76.19 ± 0.01 ^Bb^	78.68 ± 0.18 ^Cb^	83.92 ± 0.01 ^Db^	88.41 ± 0.05 ^Eb^	95.47 ± 0.08 ^Fc^
C	56.40 ± 0.09 ^Ac^	88.68 ± 0.10 ^Bc^	88.57 ± 0.05 ^Bc^	89.42 ± 0.37 ^Cc^	91.43 ±0.03 ^Dc^	92.85 ± 0.07 ^Eb^
	OH inhibition (%)					
A	22.57 ± 0.96 ^Aa^	27.53 ± 0.67 ^Ba^	46.87 ± 0.29 ^Ca^	62.22 ± 0.92 ^Da^	66.87 ± 0.83 ^Ea^	52.98 ± 0.41 ^Fa^
B	37.82 ± 0.40 ^Ab^	33.48 ± 0.57 ^Bb^	65.10 ± 0.06 ^Cb^	71.62 ± 0.22 ^Db^	85.06 ± 0.05 ^Eb^	79.09 ± 0.06 ^Fb^
C	44.95 ± 0.50 ^Ac^	41.57 ± 0.89 ^Bc^	70.56 ± 0.44 ^Cc^	72.87 ± 0.36 ^Dc^	89.07 ± 0.20 ^Ec^	83.67 ± 0.06 ^Fc^

**A**—sample with 5% (*w*/*w*) lupin seeds content, **B**—sample with 10% (*w*/*w*) lupin seeds content, **C**—sample with 15% (*w*/*w*) lupin seeds content. Values are means ± standard deviation of triplicate determinations. Means with different lowercase in the same column are significantly different at *p* < 0.05. Means with different uppercase in the same raw are significantly different at *p* < 0.05.

**Table 4:** Color values of fermented beverages and unfermented (control) samples.

Time of Storage (Days)						
Unfermented		1	4	7	14	21
L*						
A	77.45 ± 0.01 ^Aa^	81.83 ± 0.01 ^Ba^	80.03 ± 0.01 ^Ca^	81.55 ± 0.01 ^Db^	82.21 ± 0.12 ^Eb^	81.37 ± 0.02 ^Fa^
B	77.23 ± 0.02 ^Ab^	82.41 ± 0.01 ^Bc^	82.18 ± 0.02 ^Cc^	82.26 ± 0.01 ^Dc^	82.30 ± 0.02 ^Ec^	82.52 ± 0.04 ^Fc^
C	76.76 ± 0.02 ^Ac^	81.65 ± 0.02 ^Bb^	81.23 ± 0.02 ^Cb^	80.15 ± 0.02 ^Da^	81.71 ± 0.03 ^Ea^	81.64 ± 0.07 ^Eb^
a*						
A	2.51 ± 0.01 ^Aa^	2.68 ± 0.01 ^Ba^	3.55 ± 0.01 ^Cc^	2.26 ± 0.01 ^Da^	2.57 ± 0.01 ^Ea^	2.38 ± 0.01 ^Fa^
B	2.80 ± 0.01 ^Ab^	2.66 ± 0.01 ^Ba^	2.64 ± 0.01 ^Ca^	2.50 ± 0.01 ^Dc^	2.63 ± 0.02 ^Cb^	2.68 ± 0.02 ^Bb^
C	3.18 ± 0.02 ^Ac^	3.12 ± 0.01 ^Bb^	3.05 ± 0.01 ^Cb^	2.35 ± 0.01 ^Db^	3.21 ± 0.01 ^Ec^	3.07 ± 0.02 ^Cc^
b*						
A	37.15 ± 0.01 ^Aa^	31.93 ± 0.01 ^Ba^	35.85 ± 0.01 ^Cc^	30.97 ± 0.01 ^Da^	31.08 ± 0.07 ^Ea^	30.31 ± 0.01 ^Fa^
B	37.09 ± 0.01 ^Ab^	32.00 ± 0.02 ^Bb^	31.61 ± 0.01 ^Cb^	32.15 ± 0.01 ^Dc^	31.09 ± 0.02 ^Ea^	31.34 ± 0.05 ^Fb^
C	37.13 ± 0.02 ^Aa^	32.04 ± 0.02 ^Bb^	31.35 ± 0.01 ^Ca^	31.12 ± 0.02 ^Db^	31.84 ± 0.01 ^Eb^	31.48 ± 0.02 ^Fc^

**A**—sample with 5% (*w*/*w*) lupin seeds content, **B**—sample with 10% (*w*/*w*) lupin seeds content, **C**—sample with 15% (*w*/*w*) lupin seeds content. Values are means ± standard deviation of triplicate determinations. Means with different lowercase in the same column are significantly different at *p* < 0.05. Means with different uppercase in the same raw are significantly different at *p* < 0.05.

**Table 5:** Viscosity and firmness changes of the samples during storage.

	Time of Storage (Days)					
	Unfermented	1	4	7	14	21
Viscosity (MPa·s)						
A	422.57 ± 0.05 ^Aa^	433.33 ± 0.40 ^Ba^	948.00 ± 0.67 ^Ca^	481.00 ± 0.58 ^Da^	399.50 ± 1.00 ^Ea^	399.50 ± 0.28 ^Ea^
B	766.82 ± 0.40 ^Ab^	811.00 ± 0.21 ^Bb^	1030.00 ± 5.77 ^Cb^	829.00 ± 2.00 ^Db^	707.00 ± 1.73 ^Eb^	704.00 ± 4.04 ^Fb^
C	1044.95 ± 0.50 ^Ac^	1038.67 ± 0.11 ^Bc^	2846.00 ± 1.33 ^Cc^	1182.50 ± 0.50 ^Dc^	1172.50 ± 0.86 ^Ec^	1083.67 ± 0.06 ^Fc^
Firmness (N)						
A	0.19 ± 0.07 ^Aa^	0.27 ± 0.07 ^Ba^	0.39 ± 0.07 ^Ca^	0.26 ± 0.05 ^Da^	0.19 ± 0.06 ^Eb^	0.11 ± 0.04 ^Fa^
B	0.47 ± 0.04 ^Ab^	0.55 ± 0.04 ^Bb^	0.65 ± 0.07 ^Cb^	0.35 ± 0.03 ^Db^	0.18 ± 0.02 ^Ea^	0.11 ± 0.03 ^Fa^
C	0.82 ± 0.10 ^Ac^	0.90 ± 0.10 ^Bc^	1.72 ± 0.23 ^Cc^	1.14 ± 0.14 ^Dc^	1.12 ± 0.26 ^Ec^	0.70 ± 0.08 ^Fb^

**A**—sample with 5% (*w*/*w*) lupin seeds content, **B**—sample with 10% (*w*/*w*) lupin seeds content, **C**—sample with 15% (*w*/*w*) lupin seeds content. Values are means ± standard deviation of triplicate determinations. Means with different lowercase in the same column are significantly different at *p* < 0.05. Means with different uppercase in the same raw are significantly different at *p* < 0.05.

## References

[1] Aspri M., Papademas P., Tsaltas D. (2020). Review on non-dairy probiotics and their use in non-dairy based products. Fermentation.

[2] Väkeväinen K., Ludena-Urquizo F., Korkala E., Lapveteläinen A., Peräniemi S., von Wright A., Plumed-Ferrer C. (2020). Potential of quinoa in the development of fermented spoonable vegan products. LWT Food Sci. Technol..

[3] Aiello F., Restuccia D., Spizzirri U.G., Carullo G., Leporini M., Loizzo M.R. (2020). Improving Kefir Bioactive Properties by Functional Enrichment with Plant and Agro-Food Waste Extracts. Fermentation.

[4] Duranti M., Consonni A., Magni C., Sessa F., Scarafoni A. (2008). The major proteins of lupin seed: Characterisation and molecular properties for use as functional and nutraceutical ingredients. Trends Food Sci. Technol..

[5] Demir H., Simsek M., Yıldırım G. (2021). Effect of oat milk pasteurization type on the characteristics of yogurt. LWT Food Sci. Technol..

[6] Farag M., Jomaa S., El-Wahed A., El-Seedi H. (2020). The Many Faces of Kefir Fermented Dairy Products. Nutrients.

[7] Nejati F., Junne S., Neubauer P. (2020). A big world in small grain: A review of natural milk Kefir starters. Microorganisms.

[8] Dimidi E., Cox S.R., Rossi M., Whelan K. (2019). Fermented Foods: Definitions and Characteristics, Impact on the Gut Microbiota and Effects on Gastrointestinal Health and Disease. Nutrients.

[9] Hsu Y.-J., Huang W.-C., Lin J.-S., Chen Y.-M., Ho S.-T., Huang C.-C., Tung Y.-T. (2018). Kefir Supplementation Modifies Gut Microbiota Composition, Reduces Physical Fatigue, and Improves Exercise Performance in Mice. Nutrients.

[10] Silva A.R.A., Silva M.M.N., Ribeiro B.D. (2020). Health issues and technological aspects of plant-based alternative milk. Food Res. Int..

[11] Łopusiewicz Ł., Drozłowska E., Siedlecka P., Mężyńska M., Bartkowiak A., Sienkiewicz M., Zielińska-Bliźniewska H., Kwiatkowski P. (2019). Development, Characterization, and Bioactivity of Non-Dairy Kefir-Like Fermented Beverage Based on Flaxseed Oil Cake. Foods.

[12] Łopusiewicz Ł., Drozłowska E., Siedlecka P., Mężyńska M., Bartkowiak A. (2020). Preparation and characterization of novel flaxseed oil cake yogurt-like plant milk fortified with inulin. J. Food Nutr. Res..

[13] Nazhand A., Souto E.B., Lucarini M., Souto S.B., Durazzo A., Santini A. (2020). Ready to Use Therapeutical Beverages: Focus on Functional Beverages Containing Probiotics, Prebiotics and Synbiotics. Beverages.

[14] Łopusiewicz Ł., Drozłowska E., Tarnowiecka-Kuca A., Bartkowiak A., Mazurkiewicz-Zapałowicz K., Salachna P. (2020). Biotransformation of flaxseed oil cake into bioactive camembert-analogue using lactic acid bacteria, *Penicillium camemberti* and *Geotrichum candidum*. Microorganisms.

[15] Mäkinen O.E., Wanhalinna V., Zannini E., Arendt E.K. (2016). Foods for Special Dietary Needs: Non-dairy Plant-based Milk Substitutes and Fermented Dairy-type Products. Crit. Rev. Food Sci. Nutr..

[16] Jiménez-Martínez C., Hernández-Sánchez H., Dávila-Ortiz G. (2003). Production of a yogurt-like product from *Lupinus campestris* seeds. J. Sci. Food Agric..

[17] Karina T.M.-G., Uéllina S.S., Marcia R.S., Ferlando L.S., Itaciara L.N. (2018). Production of rice cereal-based Kefir beverage. Afr. J. Biotechnol..

[18] Atalar I. (2019). Functional kefir production from high pressure homogenized hazelnut milk. LWT Food Sci. Technol..

[19] Bensmira M., Jiang B. (2015). Total phenolic compounds and antioxidant activity of a novel peanut based kefir. Food Sci. Biotechnol..

[20] Corona O., Randazzo W., Miceli A., Guarcello R., Francesca N., Erten H., Moschetti G., Settanni L. (2016). Characterization of kefir-like beverages produced from vegetable juices. LWT Food Sci. Technol..

[21] Randazzo W., Corona O., Guarcello R., Francesca N., Germanà M.A., Erten H., Moschetti G., Settanni L. (2016). Development of new non-dairy beverages from Mediterranean fruit juices fermented with water kefir microorganisms. Food Microbiol..

[22] Plessas S., Nouska C., Mantzourani I., Kourkoutas Y., Alexopoulos A., Bezirtzoglou E. (2016). Microbiological Exploration of Different Types of Kefir Grains. Fermentation.

[23] Sardjono, Zhu Y., Knol W. (1998). Comparison of Fermentation Profiles between Lupine and Soybean by *Aspergillus oryzae* and *Aspergillus sojae* in Solid-State Culture Systems. J. Agric. Food Chem..

[24] Vogelsang-O’Dwyer M., Bez J., Petersen I.L., Joehnke M.S., Detzel A., Busch M., Krueger M., Ispiryan L., O’Mahony J.A., Arendt E.K. (2020). Techno-functional, nutritional and environmental performance of protein isolates from blue lupin and white lupin. Foods.

[25] Bartkiene E., Sakiene V., Bartkevics V., Juodeikiene G., Lele V., Wiacek C., Braun P.G. (2018). Modulation of the nutritional value of lupine wholemeal and protein isolates using submerged and solid-state fermentation with *Pediococcus pentosaceus* strains. Int. J. Food Sci. Technol..

[26] Bartkiene E., Bartkevics V., Rusko J., Starkute V., Bendoraitiene E., Zadeike D., Juodeikiene G. (2016). The effect of *Pediococcus acidilactici* and *Lactobacillus sakei* on biogenic amines formation and free amino acid profile in different lupin during fermentation. LWT Food Sci. Technol..

[27] Klupsaite D., Juodeikiene G., Zadeike D., Bartkiene E., Maknickiene Z., Liutkute G. (2017). The influence of lactic acid fermentation on functional properties of narrow-leaved lupine protein as functional additive for higher value wheat bread. LWT Food Sci. Technol..

[28] Martínez-Villaluenga C., Zieliński H., Frias J., Piskuła M.K., Kozłowska H., Vidal-Valverde C. (2009). Antioxidant capacity and polyphenolic content of high-protein lupin products. Food Chem..

[29] Karamać M., Orak H.H., Amarowicz R., Orak A., Piekoszewski W. (2018). Phenolic contents and antioxidant capacities of wild and cultivated white lupin (*Lupinus albus* L.) seeds. Food Chem..

[30] Awad R.A., Salama W.M., Farahat A.M. (2014). Effect of lupine as cheese base substitution on technological and nutritional properties of processed cheese analogue. Acta Sci. Pol. Technol. Aliment..

[31] Abdel–Salam A., Ali J., Zayan A. (2015). Effect of Lupine Powder on Rheological, Chemical and Microbiological Properties of Yoghurt. J. Food Dairy Sci..

[32] Khan M.K., Karnpanit W., Nasar-Abbas S.M., Huma Z.-E., Jayasena V. (2015). Phytochemical composition and bioactivities of lupin: A review. Int. J. Food Sci. Technol..

[33] Mohamed S., Awad R., Elbatawy O., Salama W. (2019). Production of vegetable yoghurt like from lupin milk. Arab Univ. J. Agric. Sci..

[34] Al-Saedi N., Agarwal M., Ma W., Islam S., Ren Y. (2020). Proteomic Characterisation of Lupin (*Lupinus angustifolius*) Milk as Influenced by Extraction Techniques, Seed Coat and Cultivars. Molecules.

[35] Zaouadi N., Hadj Ziane-Zafour A., Ouazib M., Arab Y., Hacini K., Aslan S.S. (2019). Formulation and Optimization by Experimental Design of Dairy Dessert Based on *Lupinus albus* L. and *Stevia rebaudiana* Extracts. Asian J. Dairy Food Res..

[36] Tomczak A., Zielińska-Dawidziak M., Piasecka-Kwiatkowska D., Lampart-Szczapa E. (2018). Blue lupine seeds protein content and amino acids composition. Plant Soil Environ..

[37] Hickisch A., Bindl K., Vogel R.F., Toelstede S. (2016). Thermal treatment of lupin-based milk alternatives—Impact on lupin proteins and the network of respective lupin-based yogurt alternatives. Food Res. Int..

[38] Tangyu M., Muller J., Bolten C.J., Wittmann C. (2019). Fermentation of plant-based milk alternatives for improved flavour and nutritional value. Appl. Microbiol. Biotechnol..

[39] Kasprowicz-Potocka M., Borowczyk P., Zaworska A., Nowak W., Frankiewicz A., Gulewicz P. (2016). The effect of dry yeast fermentation on chemical composition and protein characteristics of blue lupin seeds. Food Technol. Biotechnol..

[40] Zaworska A., Kasprowicz-Potocka M., Frankiewicz A., Zduńczyk Z., Juśkiewicz J. (2016). Effects of fermentation of narrow-leafed lupine (*L. angustifolius*) seeds on their chemical composition and physiological parameters in rats. J. Anim. Feed Sci..

[41] Camacho L., Sierra C., Marcus D., Guzmán E., Campos R., von Bäer D., Trugo L. (1991). Nutritional quality of lupine (*Lupinus albus* cv. Multolupa) as affected by lactic acid fermentation. Int. J. Food Microbiol..

[42] Fritsch C., Vogel R.F., Toelstede S. (2015). Fermentation performance of lactic acid bacteria in different lupin substrates-influence and degradation ability of antinutritives and secondary plant metabolites. J. Appl. Microbiol..

[43] Schlegel K., Leidigkeit A., Eisner P., Schweiggert-Weisz U. (2019). Technofunctional and sensory properties of fermented lupin protein isolates. Foods.

[44] dos Santos D.C., de Oliveira Filho J.G., Santana A.C.A., de Freitas B.S.M., Silva F.G., Takeuchi K.P., Egea M.B. (2019). Optimization of soymilk fermentation with kefir and the addition of inulin: Physicochemical, sensory and technological characteristics. LWT Food Sci. Technol..

[45] Tsafrakidou P., Michaelidou A.-M., Biliaderis C.G. (2020). Fermented Cereal-based Products: Nutritional Aspects, Possible Impact on Gut Microbiota and Health Implications. Foods.

[46] Romero-Espinoza A.M., Serna-Saldivar S.O., Vintimilla-Alvarez M.C., Briones-García M., Lazo-Vélez M.A. (2020). Effects of fermentation with probiotics on anti-nutritional factors and proximate composition of lupin (*Lupinus mutabilis* sweet). LWT Food Sci. Technol..

[47] Van Vo B., Bui D.P., Nguyen H.Q., Fotedar R. (2015). Optimized fermented lupin (*Lupinus angustifolius*) inclusion in juvenile barramundi (*Lates calcarifer*) diets. Aquaculture.

[48] Villacrés E., Quelal M.B., Jácome X., Cueva G., Rosell C.M. (2020). Effect of debittering and solid-state fermentation processes on the nutritional content of lupine (*Lupinus mutabilis* Sweet). Int. J. Food Sci. Technol..

[49] Siger A., Czubinski J., Kachlicki P., Dwiecki K., Lampart-Szczapa E., Nogala-Kalucka M. (2012). Antioxidant activity and phenolic content in three lupin species. J. Food Compos. Anal..

[50] Zhong L., Wu G., Fang Z., Wahlqvist M.L., Hodgson J.M., Clarke M.W., Junaldi E., Johnson S.K. (2019). Characterization of polyphenols in Australian sweet lupin (*Lupinus angustifolius*) seed coat by HPLC-DAD-ESI-MS/MS. Food Res. Int..

[51] Ruiz-López M.A., Barrientos-Ramírez L., García-López P.M., Valdés-Miramontes E.H., Zamora-Natera J.F., Rodríguez-Macias R., Salcedo-Pérez E., Bañuelos-Pineda J., Vargas-Radillo J.J. (2019). Nutritional and bioactive compounds in mexican lupin beans species: A mini-review. Nutrients.

[52] Arnoldi A., Boschin G., Zanoni C., Lammi C. (2015). The health benefits of sweet lupin seed flours and isolated proteins. J. Funct. Foods.

[53] Thambiraj S.R., Phillips M., Koyyalamudi S.R., Reddy N. (2015). Antioxidant activities and characterisation of polysaccharides isolated from the seeds of *Lupinus angustifolius*. Ind. Crops Prod..

[54] Liu J.-R., Chen M.-J., Lin C.-W. (2005). Antimutagenic and Antioxidant Properties of Milk–Kefir and Soymilk–Kefir. J. Agric. Food Chem..

[55] Zielińska E., Baraniak B., Karaś M. (2017). Antioxidant and anti-inflammatory activities of hydrolysates and peptide fractions obtained by enzymatic hydrolysis of selected heat-treated edible insects. Nutrients.

[56] Barac M., Vucic T., Zilic S., Pesic M., Sokovic M., Petrovic J., Kostic A., Ignjatovic I.S., Milincic D. (2019). The effect of in vitro digestion on antioxidant, ACE-inhibitory and antimicrobial potentials of traditional Serbian white-brined cheeses. Foods.

[57] Salachna P., Grzeszczuk M., Wilas J. (2015). Total phenolic content, photosynthetic pigment concentration and antioxidant activity of leaves and bulbs of selected *Eucomis* L’Hér. taxa. Fresenius Environ. Bull..

